# Mechanistic Insights into Molecular Targeting and Combined Modality Therapy for Aggressive, Localized Prostate Cancer

**DOI:** 10.3389/fonc.2016.00024

**Published:** 2016-02-16

**Authors:** Alan Dal Pra, Jennifer A. Locke, Gerben Borst, Stephane Supiot, Robert G. Bristow

**Affiliations:** ^1^Radiation Medicine Program, Ontario Cancer Institute, Princess Margaret Cancer Centre, University Health Network, Toronto, ON, Canada; ^2^Department of Radiation Oncology, University of Toronto, Toronto, ON, Canada; ^3^Integrated Center of Oncology (ICO) René Gauducheau, Nantes, France

**Keywords:** prostate cancer, radiotherapy, biomarkers, genomics, targeted therapies, molecular oncology, combined modality

## Abstract

Radiation therapy (RT) is one of the mainstay treatments for prostate cancer (PCa). The potentially curative approaches can provide satisfactory results for many patients with non-metastatic PCa; however, a considerable number of individuals may present disease recurrence and die from the disease. Exploiting the rich molecular biology of PCa will provide insights into how the most resistant tumor cells can be eradicated to improve treatment outcomes. Important for this biology-driven individualized treatment is a robust selection procedure. The development of predictive biomarkers for RT efficacy is therefore of utmost importance for a clinically exploitable strategy to achieve tumor-specific radiosensitization. This review highlights the current status and possible opportunities in the modulation of four key processes to enhance radiation response in PCa by targeting the: (1) androgen signaling pathway; (2) hypoxic tumor cells and regions; (3) DNA damage response (DDR) pathway; and (4) abnormal extra-/intracell signaling pathways. In addition, we discuss how and which patients should be selected for biomarker-based clinical trials exploiting and validating these targeted treatment strategies with precision RT to improve cure rates in non-indolent, localized PCa.

## Introduction

### The Role of RT in Localized Prostate Cancer

In 2014, it was estimated that over 233,000 men would be diagnosed with prostate cancer (PCa) in the North America leading to over 29,480 deaths (1). The prognosis and treatment of these men is currently determined by a number of different risk classification systems (2–5). All of these use combinations of the conventional risk stratifications: TNM staging, pathologic Gleason score (GS), and prostate specific antigen (PSA) level. Treatment options vary from active surveillance for indolent low-risk PCa (6) to different combinations of external beam radiotherapy (RT), brachytherapy, androgen deprivation therapy (ADT), and surgery. A comprehensive review of levels of evidence for the use of different types of treatment technologies, RT dose escalation, and the use of ADT is beyond the scope of this review, and the reader is pointed to several recent reviews in this area (7–15).

Despite the use of clinical prognostic factors and improved technological advances in radiation delivery and surgery, patients with localized PCa are at risk for local failure and occult metastases (not appreciated by current radiographic staging prior to treatment). Local recurrence after RT is thought to occur predominantly in regions bearing higher histological tumor burden (16, 17). Thus, strategies that improve both local control at the tumor site and eradicate occult metastases are required.

There is a pressing need to develop novel radiosensitizing strategies and agents to specifically target tumor cells to improve treatment outcome. Research exploiting the tumor-specific biology in relation to the normal tissue cells will reveal the Achilles heel of the most resistant tumor cells and regions. In this review we focus on approaches that combine RT with one or more agents to enhance the radiation response specifically for tumor cells. We focus on four important pathways that could influence RT outcome, including the: (1) androgen signaling pathway; (2) hypoxic tumor cells and regions; (3) DNA damage response (DDR) pathway; and (4) abnormal extra-/intracell signaling pathways. In addition, we provide an insight into which patients will benefit from this approach and how to select these patients by clinically feasible biomarkers.

## Current Molecular Prognostic Factors and Combination Treatments

Patient selection and stratification over and above the current use of clinical prognostic factors is the cornerstone for an individualized treatment with local therapy alone or combinations of local and systemic therapies (including the use of novel molecular targeted drugs). To explore this, the Radiation Therapy Oncology Group (RTOG) has completed studies on a wide range of immunohistochemical (IHC) markers (18). Tissues from Phase III RT trial (with and without ADT) were evaluated within a variety of localized risk groups. IHC-based assessment of protein overexpression for p53, p16 INK4a, Ki-67, MDM2, CYP3A4, and BCL2 were associated with adverse clinical outcomes (18) but has not yet been validated in modern-day IGRT–IMRT cohorts. Another approach is to study the somatic tumor genetics of patients based on tissues derived from pretreatment biopsies and utilizes genomics to add prognostic power for personalized medicine approaches. Indeed, recent studies from our own laboratory have implicated allelic changes in c-MYC, NKX3.1, PTEN, StAR, and HSD17B2 as adverse prognostic factors following IGRT (19–21). Novel gene signatures reflective of the underlying biology of PCa progression are also being developed in biopsy material and radical prostatectomy specimens [i.e., Myriad Genetics Prolaris Score™, Genome Health OncotypeDx™ Genomic Prostate Score, GenomeDx Biosciences Decipher™, NF-kB-activated recurrence predictor 21 (NARP21)] (22–26). The ability to analyze RNA expression on routine, archived, formalin-fixed, paraffin-embedded tissue samples is currently being developed and may provide analysis on the small amounts of tissue available from prostate biopsy specimens to help prognosticate patients prior to precision RT.

Table 1 presents a summary of some of the current biomarkers tested in PCa RT. If these prognostic markers were also predictive of efficacy for targeted drugs directed against abnormalities in cellular pathways in cancer cells, then this could lead to combining such drugs with precision RT.

**Table 1:** **Selected biomarkers tested with prostate cancer radiotherapy**.

Biomarker Reference	Treatment/follow-up time	Assay	BF	LF	DM	PCSS	OS	Comments
**(i) PROTEIN**								
**p53 overexpression**								
Grignon et al. (27)	RT vs. RT + STAD/5 years	IHC	NR	−	+	+	+	RTOG 86-10; pre-PSA era
Che et al. (28)	LTAD + RT vs. RT + STAD/5.9 years		−	−	+	+	−	RTOG 92-02; adverse for STAD
Vergis et al. (29)	RT + STAD/7 years		−	NR	NR	NR	NR	Not prognostic on MV; RT dose-escalation study
Scherr et al. (30)	RT/2.1 years		+	NR	NR	NR	NR	Adverse; see also data on BCL-2; short follow-up time
Ritter et al. (31)	RT/5.1 years		+	NR	NR	NR	NR	Adverse following conformal RT
D’Amico et al. (32)	RT + STAD/6.9 years		+	NR	NR	NR	NR	Adverse following RT ± AD

**Loss of p16INK4a**								
Chakravarti et al. (33)	RT vs. RT + STAD/8.9 years	IHC	NR	+	+	+	−	RTOG 86-10; adverse
Chakravarti et al. (34)	LTAD + RT vs. RT + STAD/6.3 years		−	−	+	+	−	RTOG 92-02; p16 expression adverse for STAD (suggests use of LTAD in p16^Hi^ cases)

**Loss of pRB**								
Chakravarti et al. (33)	RT vs. RT + STAD/8.9 years	IHC	NR	−	−	+	−	RTOG 86-10; loss of pRB adverse

**Ki-67 overexpression**								
Li et al. (35)	RT vs. RT + STAD/9 years	IHC	NR	NR	+	+	−	RTOG 86-10; High Ki-67 adverse
Khor et al. (36)	LTAD + RT vs. RT + STAD/9.3 years		NR	NR	+	+	+	RTOG 92-02; High Ki-67 adverse; see also data on MDM2
Pollack et al. (37)	LTAD + RT vs. RT + STAD/8 years		+	+	+	+	+	RTOG 92-02; High Ki-67 adverse (continuous variable)
Parker et al. (38)	SRT/6.2 years		+	NR	NR	NR	NR	High Ki-67 adverse following SRT
Cowen et al. (39)	RT/5 years		+	NR	NR	NR	NR	High Ki-67 adverse
Scalzo et al. (40)	RT/NA		+	NR	NR	NR	NR	High Ki-67 adverse

**DNA-PKcs**								
Bouchaert et al. (41)	RT	IHC	+	NR	NR	NR	NR	DNA-PKcs adverse

**MDM2 overexpression**								
Khor et al. (42)	LTAD + RT vs. RT + STAD/9.3 years	IHC	−	−	+	−	+	RTOG 92-02; also adverse when combined with Ki-67
Vergis et al. (29)	RT + STAD/7 years		−	NR	NR	NR	NR	Not prognostic on MV; RT dose-escalation study

**Bcl-2 and Bax overexpression**								
Khor et al. (43)	RT vs. RT + STAD/6.7 years	IHC	NR	−	−	−	−	RTOG 86-10; Bcl-2 and Bax not prognostic
Khor et al. (44)	LTAD + RT vs. RT + STAD/10.5 years		−	−	−	−	−	RTOG 92-02, negative Bcl-2/normal Bax adverse
Scherr et al. (30)	RT/2.1 years		+	NR	NR	NR	NR	Bcl-2 adverse, see also data on p53; short follow-up time
Vergis et al. (29)	RT + STAD/7 years		+	NR	NR	NR	NR	Bcl-2 adverse (suggests benefit with dose escalation)
Pollack et al. (45)	RT/5.1 years		+	NR	NR	NR	NR	Bcl-2 and Bax adverse on MV
Bylund et al. (46)	RT/6.4 years		NR	NR	NR	+	−	Bcl-2 related to favorable outcome

**AR CAG repeats**								
Abdel-Wahab et al. (47)	RT vs. RT + STAD/NA	flow cytometry	+	−	−	−	−	AR CAG repeats was not prognostic (suggests benefit with RT + STAD if short CAG repeats)

**COX-2**								
Khor et al. (48)	LTAD + RT vs. RT + STAD/8.9 years	IHC	+	−	+	−	−	RTOG 92-02; COX-2 expression was adverse

**STAT3**								
Torres-Roca et al. (49)	RT vs. RT + STAD/8.1 years	IHC	NR	−	+	−	−	RTOG 86-10; low levels of activated Stat3 was adverse

**VEGF**								
Green et al. (50)	RT/5.3 years	IHC	−	NR	−	+	−	VEGF was prognostic
Vergis et al. (51)	RT + STAD/7 years		+	NR	NR	NR	NR	VEGF was prognostic
Weber et al. (52)	RT vs. RT + STAD/8.1 years		−	NR	NR	NR	NR	VEGF was not prognostic

**HIF-1**								
Vergis et al. (51)	RT + STAD/7 years	IHC	+	NR	NR	NR	NR	HIF1 α was adverse
Weber et al. (52)	RT vs. RT + STAD/8.1 years		+	NR	NR	NR	NR	HIF1α expression was associated to favorable outcome

**EGFR**								
Weber et al. (52)	RT vs. RT + STAD/8.1 years	IHC	+	NR	NR	NR	NR	EGFR expression adverse

**Osteopontin**								
Vergis et al. (51)	RT + STAD/7 years	IHC	−	NR	NR	NR	NR	Osteopontin was not prognostic
Thoms et al. (53)	RT/NR	Elisa	−	NR	NR	NR	NR	Plasma osteopontin was not prognostic – OPN tested 1 year after treatment

**PKA**								
Pollack et al. (54)	LTAD + RT vs. RT + STAD/10.1 years	IHC	+	+	+	−	−	RTOG 92-02; PKA expression adverse for LTAD
Khor et al. (55)	RT vs. RT + STAD/12.2 years		+	+	+	−	NR	RTOG 86-10; PKA expression adverse

**ERG**								
Dal Pra et al. (56)	RT/6.2 years	IHC	−	NR	NR	NR	NR	ERG status was not prognostic

**(ii) DNA**								
**DNA ploidy**								
Pollack et al. (57)	RT vs. RT + STAD/9 years	Image analysis of Feulgen stained tissue sections	NR	NR	−	NR	+	RTOG 86-10; non-diploid tumors was adverse

**Cyp3A4 polymorphisms**								
Roach et al. (58)	LTAD + RT vs. RT + STAD/NA	PCR based detection	−	NR	NR	NR	−	Cyp3A4*1B polymorphism was not prognostic, regardless of race

**c-MYC ± PTEN**								
Zafarana et al. (19)	RT/6.7 years	aCGH + FISH	+	NR	NR	NR	NR	c-MYC gain alone or combined with PTEN loss was adverse

**NKX3.1 ± c-MYC**								
Locke et al. (21)	RT/6.7 years	aCGH + FISH	+	NR	NR	NR	NR	NKX3.1 haploinsufficiency alone or combined with c-MYC gain was adverse

**StAR; HSD17B2**								
Locke et al. (20)	RT/6.7 years	aCGH + FISH	+	NR	NR	NR	NR	Allelic losses of the loci containing StAR and HSD17B2 were adverse

**TMPRSS2-ERG**								
Dal Pra et al. (56)	RT/6.2 years	aCGH	−	NR	NR	NR	NR	TMPRSS2-ERG status was not prognostic

**NBN**								
Berlin et al. (59)	RT/6.7 years	aCGH	+	NR	NR	NR	NR	NBN gain predicted for decreased BF in RT, but not in RadP patients

**Toronto**								
Lalonde et al (60)	RT/6.7 years	100 loci DNA signature	+	NR	NR	NR	NR	Combined indices of genomic instability and hypoxia predict BF and early BF (≤18 months).

**(iii) RNA**								
**Myriad Genetics Prolaris Score™**								
Freedland et al. (61)	RT + ADT/4.8 years	31-gene RNA expression signature – CCP genes (RT-PCR)	+	NR	NR	NR	NR	RNA based diagnostic assay (CCP score) was prognostic after EBRT

**GenomeDx Biosciences Decipher™**								
Den et al. (62)	Post-operative RT/8 years*	22-gene RNA expression signature (gene expression microarray)	NR	NR	+	NR	NR	Genomic classifier is prognostic for distant metastasis

*Importance of biomarker: + is statistically significant (*p* < 0.05) as independent prognostic marker on multivariate analysis, − is not significant*.

*NR, not reported; BF, biochemical failure; LF, local failure; DM, distant metastasis; PCSS, prostate cancer specific survival; OS, overall survival; IHC, immunohistochemistry; LTAD, long-term androgen deprivation; PC, prostate cancer; RT, radiotherapy; SRT, salvage radiotherapy; aCGH, array comparative genome hybridization; FISH, fluorescence *in situ* hybridization; MV, multivariate analysis; NA, not available; median follow-up after radiotherapy; RadP, radical prostatectomy*.

Although several of these gene markers and signatures have demonstrated prognostic roles in small patient cohorts, many have not been validated in large-scale clinical trials of specific groups of patients (i.e., low, intermediate and high-risk PCa). Furthermore, comparison between genetic signatures has been limited; thus the best gene signature has not been identified. Future clinical trial studies should further probe these prognostic markers in larger cohorts to help optimize therapy for the individual patient.

Furthermore, once prognosticated appropriately, the best combinational therapy with RT should be better specified for the individual patient. Although a number of preclinical PCa studies have tested novel targeted agents in combination with RT, a search of MEDLINE and EMBASE databases from 2000 to 2014 shows that few of these preclinical strategies have led to the clinical trials evaluating these combinations. Instead, many of the ongoing trials are testing the use of non-targeted chemotherapies with RT in high-risk groups (Table 2). Early evidence supports this approach mainly through cytotoxic effects to micrometastatic disease and possibly addressing androgen-resistant clones. Neoadjuvant setting chemotherapy would present a synergistic role by radiosensitizing tumor cells at the primary site (63–65). The RTOG 0521 is a Phase III trial that tested adjuvant combination of docetaxel, ADT, and RT in comparison to RT and ADT in patients with high-risk localized PCa. Four-year OS rates were 89% for men who received ADT and RT vs. 93% for men treated with ADT, RT, and docetaxel (HR = 0.70; 90% CI, 0.51–0.98; *P* = 0.04). Whether adding chemotherapy will become a standard of care for this population, especially considering toxicity outcomes, remains to be seen (66).

**Table 2:** **Ongoing clinical trials testing radiotherapy combined with chemotherapy in non-indolent, localized prostate cancer**.

Agent	Study phase	Title	Protocol ID
Cabazitaxel	I	Cabazitaxel with radiation and hormone therapy for prostate cancer	NCT01420250
Cabazitaxel	II	Cabazitaxel and radiation for patients with pathologically determined Stage 3 prostate cancer and/or patients with PSA elevation (>0.1 to <2.0 ng/mL)	NCT01650285
Docetaxel	II	The ELDORADO (Eligard^®^, docetaxel, and radiotherapy) study	NCT00452556
Docetaxel	III	Treatment of prostate cancer with docetaxel + hormonal treatment vs. hormonal treatment in patients treated with radical radiotherapy (AdRad)	NCT00653848
Docetaxel	I/II	Postoperative radiation therapy, hormonal therapy, and concurrent docetaxel for high risk pathologic T2-T3N0 prostate cancer	NCT00669162
Docetaxel	II	Docetaxel, androgen deprivation, and proton therapy for high-risk prostate cancer	NCT01040624
Docetaxel	II	Docetaxel + prednisone with or without radiation for castrate-resistant prostate cancer	NCT01087580
Docetaxel	III	Androgen suppression therapy and radiation therapy with or without docetaxel in treating patients with high-risk localized prostate cancer	NCT00651326
Docetaxel	III	Hormone therapy plus radiation therapy with or without combination chemotherapy in treating patients with prostate cancer	NCT00004054
Docetaxel	III	Hormone suppression and radiation therapy for 6 months with/without docetaxel for high-risk prostate cancer	NCT00116142
Docetaxel	III	Hormone therapy with or without docetaxel and estramustine in treating patients with prostate cancer that is locally advanced or at high risk of relapse	NCT00055731
Ixabepilone	I/II	Radiation therapy and ixabepilone in treating patients with high-risk stage III prostate cancer after surgery	NCT01079793

Herein, we share insight as to how to move this area of research forward improving personalized medicine for PCa patients in this era of novel prognostic and predictive markers and targeted therapies.

## Combining Molecular Targeting and RT in PCa

### Androgen Depriving Associated Therapies and RT

#### Conventional ADT Plus RT

In the 1990s, ADT such as luteinizing hormone-releasing hormone (LH-RH) agonist or antiandrogens were tested as a combined modality therapy with RT (67). Phase III studies showed that ADT combined with RT allowed for better tumor control and survival as compared to RT alone in intermediate- and high-risk patients, and it is now considered as a standard treatment (68–70). However, despite ADT–RT combined treatments, long-term follow-up at 10 years shows that about 50% of patients relapse and eventually 10–25% die of PCa (68, 71, 72), which further strengthens the need for novel drugs especially in the high-risk category.

#### ADT Plus RT: Mechanistic Insight

The mechanism(s) of interaction between ADT and RT is still not completely clarified. An important *in vitro* study showed that different PCa cells lines lacked an overall radiosensitization by ADT (73) whereas *in vivo* data showed synergism with ADT and RT (fractionated vs. single-dose). This may be explained by the fact that the ADT effect was related to the tumor microenvironment and not to the tumor cells *per se* (74). ADT potentially affects tumor vascularization, and subsequently, tumor oxygenation. Testosterone was shown to act as a potent stimulator of prostatic endothelial cell growth (75, 76), and ADT induced a decrease in Mean Vessel Density (MVD) rapidly followed by an increase in MVD (76). Hypoxia is considered as an adverse predictive factor of RT response of prostate tumors (51, 77). ADT could decrease tumor hypoxia fraction in PCa, and this may represent a plausible explanation of the radiosensitizing properties of ADT (74). Moreover, it has been recently shown important new interactions between androgen signaling and DNA repair genes. In biopsies from patients with locally advanced PCa, androgen deprivation caused decreased levels of the Ku70 protein [responsible for non-homologous end-joining (NHEJ) repair of DNA double-strand breaks (DSBs)]; thus impairing DNA repair and possibly explaining increased radiosensitivity (78). Polkinghorn et al. (79) has recently shown that androgen receptor (AR) regulates a transcriptional program of DNA repair genes that promote PCa radioresistance. PCa cells treated with irradiation plus androgen demonstrated enhanced DNA repair and decreased DNA damage, whereas antiandrogen treatment caused increased DNA damage (also via decreased classical NHEJ) and decreased clonogenic survival. Careful monitoring of tumor vascularization, hypoxia, DNA damage markers (i.e., Ku70), the development of serum biomarkers of CYP17A1 (see below), and AR activity will be crucial to identify those patients likely to respond to ADT and RT as well as new combined modality combinations.

#### Novel Molecules Targeting Androgen Receptor Plus RT

**Figure 1:**
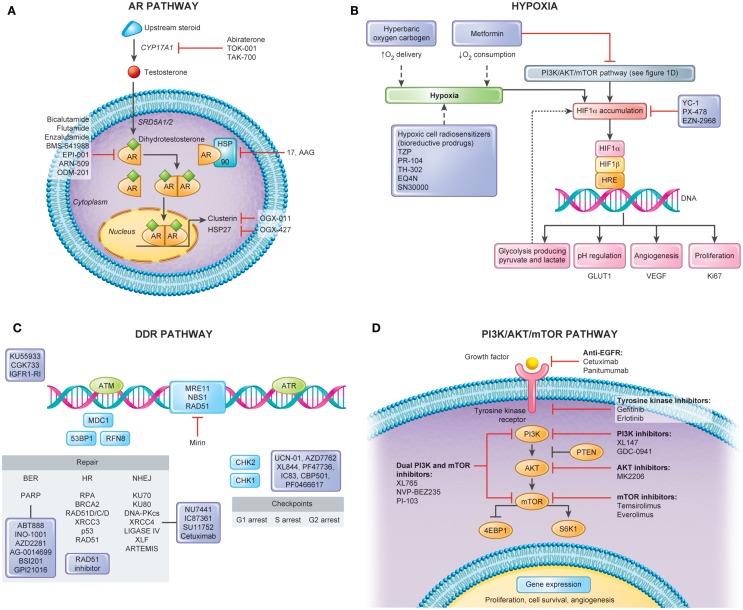
**Pathways for molecular targeting in prostate cancer radiotherapy**. Several pathways can serve as potential targets in attempt to modulate radiotherapy response and enhance clinical outcomes in non-indolent, localized prostate cancers. This figure depicts four important pathways involved in disease progression and radiation response along with its potential targets. **(A)** Androgen Receptor (AR) Pathway. AR has a central role in the transcription of several genes important in the survival and proliferation of prostate cancerous cells. Several new agents have been explored in castration-resistant prostate cancers with encouraging results. In localized disease, when combined with radiotherapy, these novel therapies also constitute a promising avenue for cure. **(B)** Hypoxia. Hypoxia modulation constitutes an important way to improve clinical outcomes following prostate cancer radiotherapy. Tumor hypoxia fraction can be targeted either by hypoxia cell radiosensitizers, enhancing oxygen delivery, or decreasing oxygen consumption. **(C)** DNA Damage Response (DDR) Pathway. Figure shows simplified DDR scheme with agents acting in different repair processes including Base Excision Repair (BER), Single Strand Break (SSB), Non-homologous End-Joining (NHEJ), and Homologous Recombination. Targeting cell cycle checkpoint can lead to cells with abrogated G1 and G2 checkpoints and accumulation of DNA breaks resulting in mitotic catastrophe. The use of these agents with RT would be expected to lead to increased residual SSBs and DSBs that can be tracked by DNA repair foci within irradiated tumor and normal tissues. In addition, the use of some of these inhibitors (e.g., PARP inhibitors) may lead to cytotoxicity as single agents based on the concept of synthetic lethality. **(D)** PI3K/AKT/mTOR Pathway. Proliferation of prostate cancer cells is under control of the PI3K/AKT/mTOR signaling. As major growth factor receptors (e.g., EGFR, VEGFR) require this downstream kinase pathway, it is also promising target for radiosensitization through inhibitors of mono- or multiple actions.

Depicted in Figure 1A is a summary of targets of the androgen axis that are currently being exploited in PCa treatment. Many of these agents have shown efficacy in castration-resistant disease. We contend that a number of the newer targeted agents could be combined with RT in localized PCa to improve outcomes. Molecules targeting the AR pathway such as abiraterone (80), TAK700 (81), or enzalutamide (82) (formerly called MDV3100) were shown to induce tumor regression even in castration-resistant disease. As compared to LH-RH agonists that only reduce circulating testosterone levels, all of these second-generation androgen agents, except enzalutamide, inhibit also paracrine and intracrine intraprostatic testosterone production, which implies a possible direct effect on PCa cells leading to more pronounced effects on the tumor microenvironment (83). Additionally, new AR inhibitors such as enzalutamide have displayed higher potency and specificity for the AR than bicalutamide and flutamide in preclinical studies and may lead to decreased side effects (84–86).

#### CYP17A1 Inhibitors Plus RT

The CYP17A1 inhibitor, abiraterone acetate, was shown to improve overall survival with minimal side effects in metastatic PCa (14.8 vs. 10.9 months, HR = 0.65) (80). CYP17A1 is an enzyme important in the synthesis of dihydrotestosterone (DHT) from cholesterol (Figure 1A) and may be targeted in the testes, adrenal glands, and prostate to reduce tumor burden in PCa. Wright et al. recently demonstrated that a SNP variant allele of CYP17A1 found in the serum of PCa patients is associated with survival (87). Furthermore, protein expression of CYP17A1 in the serum of patients with PCa is twofold higher than in the serum of healthy age-matched controls (88). These studies preclude the measurement of CYP17A1 in serum as a potential predictor for disease outcome and in light of the new CYP17A1 inhibitor, abiraterone acetate, a potential predictor for treatment response. The feasibility of circulating tumor cells (CTCs) as a biomarker of drug efficacy was recently tested and shown to be an easily obtained tissue for molecular analysis (89). It will be interesting to follow the current phase II trial of RT and ADT ± neoadjuvant or adjuvant abiraterone acetate to determine if this will be a useful means to cure intermediate to high-risk disease, and if serum CYP17A1 expression or maybe CTCs may be utilized for prediction of treatment response (NCT01023061 and NCT01780220).

TOK-001 (Galeterone) and TAK-700 (Orteronel) were shown in preclinical studies to antagonize the AR and CYP17A1 and decrease the overall expression of AR in PCa cells (90, 91). After positive results of TOK-001 in Phase II study in men with CRPC, a phase III study is planned to begin (91). TAK-700 advanced rapidly to Phase III trial in patients with CRPC; however, the study did not meet the primary endpoint of improved OS (92). Thus, further development of TAK-700 has been terminated. This affected the RTOG 1115, which was an ongoing Phase III study of dose-escalated RT with a LH-RH agonist ± TAK-700 in high-risk PCa (NCT01546987).

With the minimal side effects observed with CYP17A1 inhibitor abiraterone acetate (90), the use of these new compounds dually targeting CYP17A1 and AR are promising candidates to combine with RT.

#### Novel AR Inhibitors Plus RT

Enzalutamide is an AR antagonist with high affinity for the AR also inhibiting translocation of the AR to the nucleus and its binding to DNA. It was shown to improve OS in CRPC patients before and after chemotherapy (82, 93). In non-castrate-resistant disease, a significant biochemical response with minimal side effects was recently demonstrated in a phase II study (94). Due to important clinical response and low toxicity profile, enzalutamide is a promising drug to be utilized in the earlier stages of the disease. Possible biomarkers of enzalutamide response have been investigated and include CTCs, mutated AR, AR amplification, and AR splice variants that lack the ligand-binding domain (95–98). Detection of AR splice variant 7 messenger RNA (AR-V7) in CTCs from men with advanced disease was recently found to be associated with resistance to enzalutamide and abiraterone (98).

ODM-201 is a new generation inhibitor of the AR with superior preclinical efficacy compared to enzalutamide and bicalutamide. It does not enter the brain in preclinical studies and does not interact with cytochrome 3A4, therefore it may have lower toxicity as compared to other AR inhibitors (99).

There are several chaperone proteins associated with AR currently being targeted in CRPC including clusterin, HSP-27, and HSP-90. Serum levels of clusterin, an androgen-regulated chaperone protein, have been recently correlated with PCa outcome (100). Preclinical data has demonstrated that overexpression of clusterin decreases radiosensitivity in LNCaP cells (101) while clusterin knock-down has an effect to increase radiosensitivity of these cells (102). Moreover, a novel targeted agent to antisense clusterin (OGX-011) has been shown to be safe in men with PCa (103). Although trial data evaluating OGX-011 in patients with intermediate to high-risk PCa undergoing radical prostatectomy has been negative (104), the evaluation of OGX-011 with RT in patients with localized disease awaits investigation.

#### Androgen Depriving Associated Therapies and RT: How to Move Forward

To move forward in a personalized medicine setting, biomarker and mutation assays that reflect the functional status of the AR would help identify patients who may best benefit from these inhibitors. These assays are not in routine use in clinical RT practice, despite provocative data from the RTOG 86-10 trial that patients with short CAG repeats (which affect AR transcriptional activity) had better local control (47). The introduction of such AR biomarker methods into clinic may play an important role in the combination of current and future AR inhibitors and RT. With the introduction of such methods, second-generation antiandrogens represent interesting candidates to improve RT efficacy.

### Hypoxia and RT

The biological effects of both chronic and acute/cycling tumor hypoxia are related to increased rates of genomic instability, systemic tumor spread, and resistance to RT, and several types of chemotherapy (105, 106). Hypoxic cells when compared to oxic cells show a twofold to threefold decrease in DNA damage and cell kill after RT. The increased resistance to chemotherapy is due to decreased perfusion of agents, decreased cell kill by proliferation-dependent drugs because of hypoxic cells arrest in G0–G1 state, and increased DNA damage repair (105, 106). An increased rate of metastases is due to multiple mechanisms including increased hypoxia-activated genes involved in metastasis and angiogenesis (e.g., VEGF, LOX) and selection of potential metastatic clones during tumor progression (106).

Clinically, hypoxia has been correlated with poor clinical outcomes in PCa following RT or surgery. Turaka et al. studied 57 patients with more than 8 years of follow-up. They showed that decreased prostate-to-muscle oxygen ratio was an important predictor of early biochemical recurrence following brachytherapy (107, 108) and suggested that hypoxia was a biomarker of occult metastases at the time of treatment. Using immunohistochemistry, Vergis et al. showed that increased expression of the hypoxic markers HIF1 and VEGF leads to rapid RT failure, independent of classical clinical-pathologic factors and RT dose (51). Milosevic et al. directly measured intra-prostatic O_2_ levels of 247 PCa patients using needle–electrode technique. This was the largest clinical study of PCa hypoxia with direct measurement of tumor oxygen levels, and showed that hypoxia is associated with early biochemical relapse and local recurrence in the prostate gland (77).

#### Drugs Dependent on Hypoxia Gradient in the Tumor

Targeting hypoxia in the clinical setting has been attempted for many years (Figure 1B). This includes increasing the oxygen delivery to the tumor by the blood (normo- or hyperbaric oxygen) or the use of hypoxic cell cytotoxins, or hypoxic cell radiosensitizers. The class of agents, *N*-oxides, such as tirapazamine (TZP), is a prodrug that under hypoxic conditions undergoes intracellular one-electron reduction to highly toxic radicals that cause DSBs and DNA base damage. This damage stalls and DNA collapse replication forks. In preclinical studies, TZP is 15- to 200-fold more cytotoxic under hypoxia compared to aerobic conditions (109). The randomized phase II and III TZP studies completed to date have shown mixed tumor responses while frequently having increased normal tissue toxicity. Dinitrobenzamide mustard (DNBM) is a new class of drugs that contain a latent nitrogen mustard moiety, which becomes activated when either of the nitro groups is reduced to the corresponding hydroxylamine or amine. This results in the selective generation of reactive nitrogen mustard metabolites causing DNA cross-linking in hypoxic cells (110). PR-104, a novel DNBM currently in clinical trials has shown great promise in preclinical studies and holds several advantages over other bioreductive drugs such as TZP. First its activation is confined to lower oxygen concentrations allowing for greater specificity, and second its activated metabolites are able to diffuse locally in tumor tissue, providing an efficient bystander effect. A recent study has shown that PR-104 can be selectively active in hypoxic cells within treated 22RV1 PCa xenograft models (111). TH-302, which has a hypoxia-generated DNA damaging warhead, has also been shown to sensitize LNCaP and DU145 cells under hypoxia (112).

#### Drugs Targeting HIF-1

HIF-1 is an important transcription factor that is stabilized by low oxygen levels and is key in the expression of greater than 100 gene products following hypoxic stress. Cycling hypoxia strongly induces HIF-1, increases glucose uptake, and drives the Warburg effect. This is due in part to reoxygenation post-hypoxia increasing free radicals and thereby increasing HIF-1. HIF-1 could be a potential therapeutic target for PCa RT as it is also activated by oncogenic stress in addition to hypoxia (113). Drugs that inhibit glucose consumption by hypoxic tumor cells may be another strategy that explores the effects of hypoxia (114). This can be accomplished via HIF-1 inhibitors or by inhibition of MCT1 to force aerobic tumor cells to consume more glucose and less lactate and reduce glucose availability to the less well-perfused hypoxic cells. Lactate levels have been proposed to be a biomarker for HIF-1 inhibitors. As shown in Figure 1B, HIF-1 alpha can also be modulated by multiple upstream factors, including the PI3K/AKT/mTOR pathway (see PTEN/PI3K/AKT/mTOR) and downstream pathways, affecting gene expression, metabolism, cell survival, tumorigenesis, and tumor growth (115).

Hypoxia downregulation seems to relate to the effects of androgen deprivation in improving RT response. Al-Ubaidi et al. using pre- and posttreatment biopsies of patients treated with androgen deprivation have shown decreased HIF-1α levels by immunofluorescence (116). Many preclinical studies have tested HIF inhibition in PCa. Silencing HIF-1 alpha expression by small interfering RNA (siRNA) has shown increased radiosensitization of PC3 cells. HIF-1 alpha inhibition attenuated repair of radiation injury, with an increase in both interphase death and reproductive death after irradiation, apoptotic potential, and cell cycle arrest at the G2–M phase (more sensitive to radiation) (117). The use of dietary compounds, like soy isoflavones, has shown to improve radiation response both in PCa cell lines and xenograft models. It is believed that isoflavones inhibit the activation of the Src/STAT3 signaling pathway by radiation and radiation-induced HIF-1α expression thus contributing to increased response of cancer cells to radiation. These findings correlated with decreased expression of APE1/Ref-1 resulting in decreased DNA binding activity of HIF-1α and NF-κB, thereby inhibiting transcription of downstream genes essential for tumor growth and angiogenesis (118). Through HIF-1 abrogation and altered DNA damage repair, increased radiation response has been seen with nitric oxide donating non-steroidal anti-inflammatory drugs (NO-NSAIDs) (119). PX-478 is an oral agent that is currently under investigation in a phase I trial for advanced PCa. *In vitro*, it was shown to decrease HIF-1 alpha in PC3 and DU145 cells and enhance the radiosensitivity of PC3 cells under normoxic and hypoxic conditions (120).

#### Drugs Targeting Oxygen Consumption

While most strategies to modulate tumor hypoxia aim at increasing oxygen supply during RT through breathing of hyperbaric oxygen (121) or an oxygen-rich gas like carbogen (95% O_2_, 5% CO_2_) in combination with vasodilating agents (122), an alternative approach is decreasing oxygen consumption (Figure 1B). This is a logical choice given that oxygen gradients and “diffusion limited” hypoxia arise due to high cellular oxygen consumption (123). Mathematical modeling suggests that decreasing oxygen consumption is more efficient at promoting tumor oxygenation than increasing oxygen supply (124).

Our group has investigated metformin, a commonly prescribed anti-diabetic drug, as an effective and inexpensive means to improve RT outcome. Metformin inhibits complex I activity in the mitochondrial electron transport chain (ETC), therefore inhibiting cellular oxygen consumption (125). We showed through *in vivo* and *in vitro* models that metformin could improve tumor radiation response through inhibiting tumor cell oxygen consumption and transiently increasing tumor oxygenation. We also tested the impact of metformin use on the outcome of 504 PCa patients treated with curative-intent RT. Metformin was associated with an independent and significant decrease in early biochemical relapse rates (126). Others groups have confirmed the clinical benefit of metformin in PCa patients undergoing RT (127, 128).

#### Targeting Hypoxia: How to Move Forward

In order to personalize combined therapy approaches in PCa, clear and accurate documentation of preexisting and/or treatment-induced aggressive/adaptive tumor microenvironments is required to tailor such treatment to patients. Any trials with hypoxia-modifying agents will require biomarkers that measure hypoxic fraction before and after modification to place hypoxic patients into appropriate trials and prove that the drug is active in hypoxic tumor subpopulatons. Currently, different methods of hypoxia measurement have been used including pO_2_ microelectrodes, *in situ* markers including extrinsic markers (EF-5 and pimonidazole) or intrinsic markers (e.g., HIF1, VEGF, and GLUT-1) and imaging modalities involving functional PET and MRI (129). If hypoxia is to become a criterion for disease management in PCa, an agreement on invasive and/or non-invasive biomarkers is notably required (105, 130, 131).

Existing technologies can deliver a higher RT dose to specific regions in the tumor (i.e., dose painting) without increasing the dose in surrounding normal tissue. Dose painting as function of non-invasive hypoxia imaging modalities in combination with hypoxia-targeted systemic agents may be the way forward (132); as the prognostic value of low pO_2_ and increased expression of hypoxia-associated markers *in situ* was shown to be independent of radiation dose (51, 77), the hypoxic sub-fraction may therefore benefit from both dose-escalation and systemic treatment.

### Targeting DNA Damage Responses and DNA Repair

#### DNA Damage Responses and DNA Repair in RT

Radiation therapy results in the production of a variety of ionizing radiation-induced lesion in DNA. Specific pathways of DNA repair are required to repair the variety of lesions, which include DNA single-strand breaks (SSBs), DSBs, DNA base alterations, and DNA–DNA or DNA–protein cross-links. Non-repaired DNA damage can lead to normal and tumor cell death via apoptosis, mitotic catastrophe, autophagy, or terminal growth arrest senescence. In PCa patients, RT was shown to induce ATM-p53 DNA damage-dependent proteins thereby leading to long-term activation of p21WAF associated with reduced cell proliferation, but no apoptosis (133). Precise molecular targeting of the sensing and repair of DNA damage in PCa cells over surrounding normal tissues (e.g., rectum, bladder, bowel) is a promising area of combination therapy in non-indolent, localized PCa.

DNA DSBs are the most damaging breaks resulting in cell death. DNA DSBs are primarily repaired through two different pathways: HR and NHEJ. HR repair is a template-guided, error-free pathway predominantly operating in the S and G2 phases of the cell cycle, which express many HR-related proteins, including Rad51, the Rad51 paralogs (XRCC3, RAD51B,C.D) XRCC2, RPA, BRCA2, and BLM proteins. In contrast, NHEJ is operational in all phases of the cell cycle and uses the KU 70/80, DNA-PKcs, Artemis, XLF, XRCC4, and DNA ligase IV proteins. The latter pathway would therefore only be operational in G0/G1-arrested slowly proliferating, late-reacting tissues (which limit the total dose of fractionated RT) (134).

#### Approaches to Target DNA Damage Responses

##### Using the Genetic Defects in Tumor Cells Involved in DNA Damage Response

Strategies that target DNA repair pathways that are dependent on DNA replication (i.e., HR during the S phase) may give rise to a therapeutic ratio when combined with fractionated RT (Figure 1C). We have shown that Rad51 expression and functional HR can be reduced using imatinib in PCa cells *in vitro* and *in vivo* during experimental RT (135). This combined imatinib–RT treatment increased prostate tumor cell radiosensitization without increased gut toxicity. Similar preclinical data exist *in vitro* for the targeting of the SSB and BER repair pathways [e.g., inhibiting the activity of poly (ADP-ribose) polymerase (PARP) or DNA polymeraseβ] whereby the increased levels of non-repaired SSB are converted to more lethal DSBs during replication. As such, the differential targeting of DNA repair in replicating tumor cells vs. non-replicating late-reacting normal tissues could be exploited in clinical treatment protocols.

Synthetic cell lethality defines a genetic interaction in which the combination of mutations in two or more genes (each mutation on its own being non-toxic) leads to cell death. A number of lethal combinations have been discovered using silencing RNA (siRNA) and chemical screens and, subsequently, validated in isogenic preclinical model systems and phase I and II clinical trials. From these screens, it was observed that certain DNA repair inhibitors may lead to tumor cell kill when used as single agents as they cause synthetic cell lethality when combined with a germ-line or somatic genetic defect in DNA repair. A remarkable example of this interaction is the results of recent trials that have observed tumor responses in chemoresistant breast and ovarian cancers with HR defects (BRCA1/2 deficient and HR defective) using inhibitors of the SSB repair protein, PARP1 without toxicity to repair-proficient normal tissues (136). Other approaches are to use PARP inhibitors with tumors deficient in phosphatase and tensin homolog (PTEN), Aurora A kinase, and HR- or cell cycle-related pathways or using DNA polymerase-β inhibitors in mismatch repair deficient tumors. These synthetic lethality approaches can be designed to decrease the number of PCa clonogens prior to RT if used in a neoadjuvant fashion and improve RT outcome (134).

DNA repair enzyme inhibition (e.g., PARP inhibition) may be prolonged in tumor tissues relative to normal tissues *in vivo* and recent data suggest that PARP inhibitors can “trap” the PARP1 and PARP2 enzymes at damaged DNA (137). In the latter scenario, the pharmacodynamics of an oral or intravenous inhibitor could determine when RT is administered during the period when tumor enzymes are still inhibited for DNA repair function, yet the pathway is no longer inhibited in normal tissues. Careful pharmacodynamic studies may lead to an increased therapeutic ratio based on differential scheduling of fractionated RT with a DNA repair inhibitor. Knowledge *a prior* of germ-line and somatic mutations in DNA damage and repair genes in RT patients could therefore be very helpful if the mutations lead to a functional loss of specific response pathways. We have used array comparative genomic hybridization to show that there can be allelic loss of PARP1, ATM, DNA-PKcs, p53, Rb, and RAD17 in PCa (59). If this leads to functional loss of DNA repair or damage signaling, then these patients may benefit from targeted therapies (e.g., inhibitors of PARP, ATM, DNA-PKcs, MTp53, and CHK1) in addition to the potential tumor cell radiosensitization based on inherently abnormal DNA repair (105).

##### Using the Difference of Cell Cycle Phase Stages between Tumor and Normal Cells

The radiosensitivity of human cells varies throughout the cell cycle (i.e., G1, S, G2, and M phases). S-phase cells are relatively more radioresistant than G1 and G2/M cells. Tumor cells have a shorter interval of subsequent cell cycles with a higher S-phase fraction correlated with base excision repair and/or homologous recombination (HR) compared to late acting G1-arrested normal tissue cells. Therefore the use of inhibitors of HR such as a recently described RAD51 inhibitor (138) may be selective for tumor cells over late reacting normal tissues.

##### Targeting Hypoxia Related Differences in DNA Damage Repair Pathway in Tumor Cells

Prolonged acute or chronic hypoxia can lead to decreased expression of HR genes, which decreases the radioresistance (e.g., reduced oxygen enhancement ratio); HR-deficient hypoxic cells can then be more radiosensitive when reoxygenated than even HR-proficient oxic cells (139). Thus, although acutely anoxic tumor cells that are repair proficient may be highly resistant to ionizing radiation, chronically hypoxic tumor sub-regions may contain cells with differential radio- and chemosensitivity. We observed that HR-defective hypoxic cells are more sensitive to radiation, mitomycin C, and cisplatin (140). Furthermore, these repair-deficient cells may also be more sensitive to PARP inhibitors; a phenomenon termed “contextual synthetic lethality” (141). Clinically useful functional assays of repair-proficient vs. repair-deficient oxic and hypoxic cells will be required to show the fraction of repair-deficient hypoxic cells in a given tumor. This could be useful to tackle advantage of contextual synthetic lethality using molecular targeted inhibitors with RT (142).

### Abnormal Extra-/Intracell Signaling

#### PTEN/PI3K/AKT/mTOR

Proliferation of PCa cells is under control of the PTEN/PI3K/AKT protein pathways (143, 144) (Figure 1D), and this is also critical for PCa stem-like cell maintenance (145). Loss of PTEN, a common event in many human cancers, can be detected in more than 60% of PCa. This leads to constitutive activation of AKT and thereby activation of a host of downstream proteins that are involved in cell cycle progression, apoptosis suppression, and glucose uptake and metabolism (146). Permanent AKT activation is a major factor of radioresistance and is an important target to increase the RT response (147, 148). Also, interactions between different extra- and intra-cell signaling pathways play a significant role in radioresistance.

In addition, signal transduction modulation interferes with DNA repair mechanisms, particularly DSB repair by NHEJ (147) being an important alternative to increase radiosensitivity. PI3K-dependent AKT phosphorylation triggers a downstream cascade of events that are likely to interact with AR transcriptional activity. These include interaction of the AR with FKHR and FKHRL1 transcription factors, cross-talk of AR and AKT with NF-κβ, regulation of AR via coactivator Wnt/β-catenin, and activation of AR via the mTOR pathway (147). PI3K/AKT/mTOR downstream kinase pathways also regulate NF-κB which, in turn, regulates AR expression (149) and various other pathways implicated in cell survival, proliferation, invasion, angiogenesis, and metastasis (112). Numerous agents identified from natural sources can block the NF-κB pathway, including curcumin, resveratrol, ursolic acid, capsaicin, silymarin, guggulsterone, and plumbagin. Curcumin was shown to downregulate both the NF-κB and Stat3 pathways (149–153).

Pharmacological mTOR inhibition has been demonstrated to block the induction of the proliferative, pro-survival, and oncogenic functions of mTOR (154), with important effects in PTEN-deficient tumors. mTOR signaling has been implicated as a determinant of cell survival in response to DNA damage (155). mTOR inhibitors have been shown to potentiate the effects DNA damaging agents, including ionizing radiation (156–159). As such, the mTOR-signaling pathway is a promising target for RT optimization in PCa.

Rapamycin, when administered in localized PCa patients before prostatectomy, attained high intra-prostatic levels with minimal adverse effects and effectively limited mTOR signaling. This was determined by inhibition of S6 kinase phosphorylation, which is a downstream target of mTOR activity involved in protein translation (160). Although some preliminary results with mTOR inhibitors (temsirolimus and everolimus) have been disappointing when administered as single agents in castration-resistant disease, they showed radiosensitizing effects independent of castration status (156). The combination of RAD001 with radiation has been tested in phase I and II trials (NCT00657982, NCT01548807, NCT00943956). Dual PI3K/mTOR inhibitors (BEZ235 or PI103) when combined with RT greatly improved treatment efficacy by repressing colony formation, inducing more apoptosis, leading to the arrest of the G2/M phase, increased double-strand break levels, and less inactivation of cell cycle check point, autophagy and NHEJ/ HR repair pathway proteins in PCa-radioresistant cells (161). BEZ235 has been shown to improve tumor sensitization by improving tumor oxygenation and vascular structure (162–164), and the radiosensitizing properties of BEZ235 seem to occur in normoxic and hypoxic PCa cells (164). Other PI3K inhibitors like XL147, GDC-0941, XL765, and small-molecule AKT inhibitors MK2206 are currently in Phase I trial (144) and are promising candidates for future studies with RT. Predictive biomarkers are essential for the clinical success of these agents targeting the PI3K/AKT/mTOR pathway. Possible biomarkers for mTOR inhibitors response may be phosphorylated p70S6K, pS6, AKT as well as VEGF, BCL2, and PTEN.

The Akt inhibitor Erufosine (ErPC3) was studied in PCa cell lines. It was shown to have a potential therapeutic benefit when used as monotherapy or in combination with RT (165). The Akt inhibitor P529 potentiates the effect of RT in PC3 cells mainly not only through the blockade of Akt activation but also through the alteration of other cancer-related pathways involving MMP-2, MMP-9, Id1, and VEGF. P529 also enhances the antitumor effect of RT *in vivo* by reducing the proliferation rates and promoting apoptosis. This ability to act at different pathway levels, all of them involved in the response to radiation, makes this compound an interesting agent for radiosensitization (166).

#### EGFR

EGFR for a long time has been considered an appealing target for monoclonal antibody (mAb) therapy. The treatment of head and neck cancers with EGFR inhibitors represents a model for the optimization of RT with molecular targets (167–169). In non-metastatic PCa, studies have reported high EGFR expression ranging from 18 to 41% (170, 171). There is evidence that the activation of EGFR and downstream signaling pathways are implicated in cell survival and proliferation following radiation (172–174) thus several studies have addressed potential mechanisms for radiosensitization by EGFR inhibitors. The prognostic role of EGFR expression in PCa is not clearly defined, although some studies have shown that an increased EGFR expression was associated with higher GS, early PSA relapse, and progression to CRPC (170, 175–178).

Exposure of tumor cells to radiation results in immediate activation of EGFR by autophosphorylation (179) and a secondary prolonged release of TGF-α (180). This creates an autocrine loop, which is important for proliferation and is thought to play a part in accelerated repopulation following radiation (181). EGFR activation of downstream pathways including the Ras/Raf/MAPK and STAT3 pathways results in protection from radiation induced cell death (182, 183). EGFR inhibition in different model systems has shown to affect proliferation, angiogenesis, and cell survival. Radiosensitization by EGFR inhibition seem to involve changes in cell-cycle arrest, endothelial cell sensitivity, apoptosis and DDR (184, 185).

Phase I and II trials tested the EGFR inhibitor gefitinib in combination with PCa RT (186). The toxicity profile of the combination appears to be acceptable, as less than 10% of patients had toxicity-related interruptions of RT. The preliminary efficacy seems promising compared to matched controls treated with a slightly higher biologically effective dose; however, further studies are required.

When cetuximab was tested in DU145 cells, it increased the radiosensitivity through antiproliferative effect, inhibition of clonal growth, G(0)/G(1) phase arrest, apoptosis induction, and inhibition of EGFR-signaling pathways by the downregulation of MAPK activation (187). The simultaneous blockade of EGFR and VEGFR (i.e., AEE788) has been tested with radiation and can lead to significant tumor growth delay in DU145 cells (188–190). Potential mechanisms of action could include: (1) enhanced tumor vasculature destruction and (2) decreased proliferation of tumor cells surviving cytotoxic effects of RT (191). Preclinical studies with PCa cells using coinhibitors of both EGFR and type 1 insulin-like growth factor receptor (IGF1R) significantly dampened cellular growth and DDR, therefore increasing radiosensitivity. The synergistic effect of the EGFR and IGF1R inhibitors was also confirmed in nude mouse xenograft assays, thus may provide a therapeutic rationale to be tested in future clinical trials (192).

#### Immune Checkpoint Inhibitors

Experimental data from multiple cancer models have provided cumulative evidence of an interaction of ionizing radiation with the systemic antitumor immunity, and this has created several opportunities in the field (193).

The combination of immunologic checkpoint inhibitors with RT offers an additional area to improve cancer cell kill in PCa (194). Based on preclinical data, manipulating immune response through checkpoint molecules using mAbs has thus gained interest (195). Early phase I and II clinical trials have demonstrated favorable safety profiles with cytotoxic lymphocyte antigen-4 (CTLA-4) blockade via the mAbs ipilimumab and tremelimumab (196–200). Recent phase I/II trials have been conducted combining single-dose RT concomitant or sequential to ipilimumab. These trials have confirmed the preclinical data that RT could help prime an immune response (200). A phase III study subsequently compared ipilimumab with a placebo following RT (8 Gy in one fraction) and demonstrated a significant PFS benefit but no benefit in terms of OS (201).

Future clinical trials are further investigating the ability of immunologic checkpoint inhibitors to enhance RT’s effect on tumor growth rate kinetics and cellular apoptosis on clinical endpoints such as PFS and OS. Clinical studies with novel immune strategies must include tissue and blood for interrogation of how and which immunologic populations can benefit from this approach.

## Concluding Remarks and Future Perspectives

Despite all technological advances in RT delivery over the recent years, improvements in molecular characterization of PCa have not changed clinical practice. Decision-making in RT for PCa treatment is still guided by conventional clinical-pathological factors: PSA levels, GS and T category. In order to minimize RT failures (local and systemic) in non-indolent PCa, precision RT needs to exploit the rich molecular landscape of PCa.

Although the present review focused on four major pathways, other intricate and dynamic mechanisms related to intrinsic and/or acquired radioresistance contribute to the complexity of PCa radioresistance. Many groups have investigated the role of prostate cancer stem cells in providing a reservoir of cells resistant to radiation (202–205). The inhibition of signaling pathways in combination with RT may be a strategy to target PCa stem cells leading to better outcomes (205, 206) by providing improved local control and preventing the dissemination of resistant, proliferating stem-like PCa cells (207, 208). The specific role of prostate cancer stem cells has been recently reviewed (209). Another important feature in PCa radioresistance is the presence of neuroendocrine cells. Although rare, a neuroendocrine phenotype may be present at diagnosis and/or arise during the different stages of disease progression leading to a castrate-resistant state (neuroendocrine cells lack AR expression) and a lethal outcome. Although preclinical data have suggested that radiation can induce neuroendocrine transdifferentiation in PCa cell lines (210–212), an enhanced molecular identification of neuroendocrine cells with a better knowledge of the clinical impact of treatment-induced neuroendocrine differentiation are mostly warranted (211).

The optimization of PCa RT must take into consideration the importance of tumor heterogeneity. Elucidating those specific molecular processes of tumor progression in combination with a better characterization of tumor microenvironment and, most importantly, identifying and validating predictive biomarkers of treatment response are critical steps.

A significant progress has been made in the discovery of molecularly targeted therapies directly and indirectly involved in pathways of PCa progression and RT response. However, the lack of clinical trials combining RT and novel molecular agents faces several challenges. Enhancing the interest of pharmaceutical industry toward RT-based drug development and expediting the testing of these agents with RT are imperative to accelerate the field and improve patient outcomes (213).

The technological advances and the lower costs in genomic medicine should help standardize and validate assays for molecular characterizations. This is determining personalized PCa genomics and reflecting specific patterns of tumor progression. Incorporation of this knowledge into biomarker-based prospective clinical trials will enable us to marry precision RT, novel targeted agents, and biological endpoints in order to improve cure rates in non-indolent PCa.

## Author Contributions

All authors contributed equally to researching data, discussing the content, writing the article, and performing review/editing of the manuscript before submission.

## Conflict of Interest Statement

Alan Dal Pra has previously received honoraria or research grants from AstraZeneca, Astellas and Bayer. Robert G. Bristow has previously received honoraria or preclinical research grants from Abbott Pharmaceuticals, SuperGen, AstraZeneca, MERCK, and Sentinel. Stephane Supiot has previously received honoraria and research grants from Janssen, AstraZeneca, Novartis. The other co-authors declare that the research was conducted in the absence of any commercial or financial relationships that could be construed as a potential conflict of interest.
